# Mediator is an intrinsic component of the basal RNA polymerase II machinery *in vivo*

**DOI:** 10.1093/nar/gkt701

**Published:** 2013-08-20

**Authors:** Thierry Lacombe, Siew Lay Poh, Régine Barbey, Laurent Kuras

**Affiliations:** Centre de Génétique Moléculaire, Centre National de la Recherche Scientifique, affiliated with Université Paris-Sud, Gif-sur-Yvette 91198, France

## Abstract

Mediator is a prominent multisubunit coactivator that functions as a bridge between gene-specific activators and the basal RNA polymerase (Pol) II initiation machinery. Here, we study the poorly documented role of Mediator in basal, or activator-independent, transcription *in vivo*. We show that Mediator is still present at the promoter when the Pol II machinery is recruited in the absence of an activator, in this case through a direct fusion between a basal transcription factor and a heterologous DNA binding protein bound to the promoter. Moreover, transcription resulting from activator-independent recruitment of the Pol II machinery is impaired by inactivation of the essential Mediator subunit Med17 due to the loss of Pol II from the promoter. Our results strongly support that Mediator is an integral component of the minimal machinery essential *in vivo* for stable Pol II association with the promoter.

## INTRODUCTION

Mediator is a large multisubunit complex, conserved throughout eukaryotes, that plays an essential role in transcription of protein-encoding genes. It was first discovered in yeast due to its ability to support activator-driven transcription on naked DNA templates in a cell-free system reconstituted with purified RNA polymerase II (Pol II) and general transcription factors (GTFs; including TATA-binding protein (TBP), Transcription Factor (TF) IIB, TFIIF, TFIIE and TFIIH) (1). Mediator was shown to interact with numerous yeast and mammalian gene-specific transcription activators as well as with Pol II and GTFs, supporting a role as a bridge between gene-specific regulators bound at enhancers and the basal Pol II machinery assembled at the core promoter (2). Electron microscopy analyses combined with biochemical and genetics data have led to a model of topological organization in which the 25 subunits forming the yeast Mediator complex are distributed into four distinct modules named as head, middle, tail and CDK8 (3). The head, middle and tail modules constitute the core Mediator. In the presence of Pol II, the core Mediator assumes an elongated shape and makes multiple contacts with Pol II through the head and middle modules (4). The CDK8 module, which comprises a cyclin–kinase pair, associates reversibly and under specific conditions with the core complex and is mainly involved in negative regulations (5,6).

A widespread model assumes that Mediator is recruited to promoters by gene-specific activators bound to enhancer elements before binding of Pol II and GTFs (2). This model is supported by various studies, notably in yeast, showing spatial, temporal and physical separation between Mediator and Pol II recruitment at some promoters (7–9). Mediator tail module is viewed as a major target of gene-specific activators because of the numerous interactions evidenced between subunits of this module and various activators both in yeast and in mammals; however, a number of interactions involving subunits of other modules were also reported (10,11). A primary key role of Mediator once recruited by activators would be to facilitate the recruitment of GTFs and Pol II to form a pre-initiation complex (PIC). Studies using electron microscopy and single-particle-reconstruction techniques have shown that human Mediator undergoes conformational shifts on binding of activators to its tail module, supporting an allosteric model in which activators would trigger a wave of remodeling within Mediator ultimately stabilizing its association with Pol II and impacting assembly and activity of the PIC (12–14). Considering the biochemical stability of the ternary complex between Mediator, Pol II and TFIIF (13,14), an alternative possibility could be that Mediator is recruited as a larger, holoenzyme-like complex containing also Pol II. The two models are not exclusive in view of the results by Esnault *et al.* (15), suggesting multiple pathways for recruitment of Pol II and formation of the PIC *in vivo*.

In addition to its well-documented role in supporting activated transcription, Mediator was also shown to stimulate basal, or activator-free, transcription in both yeast and mammalian *in vitro* systems (16–19). This observation, together with the fact that inactivation of Med17 (also known as Srb4) impairs transcription of the majority of protein-encoding genes in yeast (6), has been taken into account as evidence that Mediator functions as a GTF, comparable in importance with Pol II and other basal factors for transcription initiation (19). However, there is no evidence to date that Mediator can bind promoter DNA and function independently of activator *in vivo*. Genome-wide localization studies using chromatin immunoprecipitation coupled with microarrays were carried out in yeast, but the results were conflicting and it has been unclear whether Mediator is always present at active promoters (20–23). Interpretation of these studies is also complicated by the strong possibility that formaldehyde does not crosslink Mediator at enhancer and core promoter sequences with the same efficiency. Therefore, the question of knowing whether Mediator is an intrinsic component of the basal machinery required for transcription initiation by Pol II *in vivo* remains unresolved. To address this issue, we have carried out ‘activator bypass’ experiments in yeast using molecular tools designed for recruiting the transcription machinery to a test promoter in the absence of classical activator, in this case through artificial tethering of a GTF (24,25). We have used these tools to determine whether Mediator is recruited alongside the Pol II machinery and is still required for transcription in this context. Our results show that Mediator is indeed found at the promoter when the Pol II transcription machinery is recruited through tethering of TFIIB or TBP. Moreover, we show that inactivation of the head module impairs activator-independent transcription driven by artificial recruitment of TFIIB or TBP. Altogether, our results provide evidence that Mediator is an integral part of the basal Pol II transcription machinery *in vivo* and functions as a general transcription initiation factor.

## MATERIALS AND METHODS

### Yeast strains and media

Genotypes are given in Supplementary Table S1. Strains expressing the chromosomal *SUA7* allele under the control of the *GAL1* promoter were generated by integrating the *KanMX6-PGAL1* cassette from Longtine *et al.* (26) upstream of the coding sequence of *SUA7* using standard procedures. Y805 was generated from W303-1A. Y807, Y809 and Y811 were generated from CL18, CL7 and DY3168, respectively, described in Leroy *et al.* (27). Y822 and Y823 were generated from Y400 and Y402, which contain a null allele of *MED17(SRB4)* on the chromosome and plasmids RY2844 (*SRB4+*, *CEN*, *LEU2*) or RY2882 (*srb4-138*, *CEN*, *LEU2*) in the W303-1A genetic background (6). Y892 was generated from Y14, which corresponds to W303-1A with an amino terminal HA-tagged TBP at the chromosome (28). Y893 and Y894 were generated from Y80 and Y84, which contain a *TAP-KlTRP1* cassette inserted before the stop codon of Med5 or Med14, respectively (9). Y909 and Y911 were generated from two isogenic strains containing either the wild-type *RPB1* allele or the *rpb1-1* allele at the chromosome, described in Nonet *et al.* (29). Y959 and Y960 were generated from two isogenic strains containing either the wild-type *KIN28* allele or the *kin28ts3* allele at the chromosome, described in Valay *et al.* (30). Y963 was generated from a *met4Δ::TRP1* strain, in which nucleotides 180–1848 of *MET4* open reading frame (ORF) were replaced by *TRP1*. This strain was crossed with Y84 and appropriate segregants combining *met4Δ::TRP1* and *MED14-TAP-klTRP1* were selected by tetrad analysis and confirmed by polymerase chain reaction (PCR).

YNB medium contains 0.7% yeast nitrogen base, 0.5% ammonium sulfate and 2% glucose. Complete Supplement Mixture (CSM) medium contains, in addition, a CSM amino acid drop-out mixture.

### Plasmids

pRS313-TFIIB-RFX (*HIS3*, *CEN*) was generated from pRS314-TFIIB-RFX (*TRP1*, *CEN*) (a gift from Michel Strubin) by subcloning an Xba1-Xho1 fragment containing the promoter region of TBP followed by the ORFs of TFIIB and the human regulatory factor X (RFX) protein. A short linker coding for the nuclear localization signal (NLS) of SV40 and the HA epitope from influenza virus is found in between TFIIB and RFX. YCp91-LexA (*CEN*, *TRP1*) contains the entire LexA coding sequence (residues 1–202) under the control of the *ADH1* promoter, followed by SV40 NLS, an HA epitope and the *CYC8* terminator (24). YCp91-LexA-TBP (*CEN*, *TRP1*) contains the *SPT15* coding sequence and terminator in place of the HA epitope (24). pRS315-LexA was generated by subcloning into pRS315 a BamH1-Sac1 fragment from YCp91-LexA containing *ADH1* promoter, SV40 NLS, HA epitope and *CYC8* terminator. pRS315-LexA-TBP was generated by subcloning into pRS315 a BamH1 fragment from YCp91-LexA-TBP containing *ADH1* promoter, SV40 NLS and SPT15 coding sequence and terminator. YCplac33-xMET17-GFP (*URA3*, *CEN*) was constructed by cloning into YCplac33-GFP (a gift from Dieter Kressler) a PCR fragment spanning nucleotides −1 to −400 of *MET17* and containing at position −200 the sequence CAGTTGCCTAGCAACTACATATGGTCACC (refer-red to as X box) including the RFX binding site found in the polyomavirus enhancer (underlined). The *xhis3* fragment in YCplac33-*xhis3-GFP* originates from a *LexAop-his3* construct containing a LexA operator inserted into a derivative of *his3* lacking the T_c_ element and a functional Gcn4 binding site (31). It was generated by replacing the LexA operator by the X box using PCR. YCplac33-*xPHO5-GFP* and YCplac33-*xGAL1-GFP* (*URA3*, *CEN*) were constructed by cloning into YCplac33-*GFP* PCR fragments spanning nucleotides −1 to −392 of *PHO5* or nucleotides −1 to −500 of *GAL1*, and containing the X box inserted at position −167 and −200, respectively. YCplac33-*LexAop-MET17-GFP* was constructed by cloning into YCplac33-*GFP* a fragment, engineered by PCR, containing the sequence CTACTGTATGTACATACAGTAGTTTGTT (LexA operator underlined) inserted at −200 in a *MET17* fragment spanning from −1 to −400 (relative to ATG).

### Chromatin immunoprecipitation (ChIP)

Cell fixation, chromatin preparation and immunoprecipitation were performed essentially as described previously (27). In Figures 2 and 7A, formaldehyde fixation was performed at 37°C to maximize crosslinking efficiency, and cells were shifted to 37°C for 60 min before adding formaldehyde. Pol II was immunoprecipitated using the rabbit polyclonal antibody y-80 (Santa Cruz Biotechnology) directed against amino acids 1–80 of the Rpb1 subunit. Typically, 10 µl of antibody was incubated overnight at 4°C with crosslinked chromatin extracted from 10 ml of culture at OD_650_ = 1. TAP-tagged proteins were immunoprecipitated by incubating the same amount of crosslinked chromatin with 30 µl of rabbit Immunoglobulin G (IgG)-agarose (Sigma) for 4 h at 4°C. DNA was quantified by real-time PCR using the LightCycler 480 instrument (Roche), and SYBR Premix Ex TaqTM (Takara). Sequences of primers are given in Supplementary Table S2. A typical run included duplicates of each IP and input DNA, and serial dilutions of one input DNA to create a standard curve and determine the efficiency of the amplification. Data were analysed with the LightCycler 480 software using the ‘second derivative maximum’ method for quantification. The level of occupancy at a specific DNA locus is calculated as the percentage of DNA present in the immunoprecipitate relative to the total input. Relative occupancy was obtained by normalizing to occupancy at the *IME2* locus.

### RNA analysis

Total RNA was extracted with hot acidic phenol following a protocol derived from Schmitt *et al.* (32). Briefly, cells collected from 10 ml of culture at OD_650_ = 0.5–1 were resuspended in 400 µl of cold AE buffer (50 mM sodium acetate, pH 5.3; 10 mM ethylenediaminetetraacetic acid, pH 8; 10% sodium dodecyl sulphate) and the suspension was mixed with 400 µl of cold phenol saturated with 0.1 M citrate buffer pH 4.3 (Sigma). The mixture was incubated for 8 min at 65°C with agitation, quickly frozen in liquid nitrogen, incubated again at 65°C with agitation for 4 min and centrifuged at room temperature for 10 min, 12 000 rpm. The aqueous phase was extracted once with 1 vol acidic phenol/chloroform (1:1), once with 1 vol chloroform and was precipitated with 0.1 vol 3 M LiCl and 2.5 vol absolute ethanol. After centrifugation, the RNA pellet was washed with absolute ethanol, air-dried and resuspended in water.

Reverse transcription (RT)-quantitative PCR was conducted following a two-step procedure using the RevertAid H Minus M-MuLV reverse transcriptase (Thermo Scientific) and random hexamers for priming, and the SYBR Green I Master mix and LightCycler 480 system (Roche) for real-time PCR. Sequence of primers is given in Supplementary Table S2.

Primer extension was performed as follows. Ten microliters containing 10 µg of RNA, 1 pmol of ^32^P-5′-labelled gene-specific oligonucleotides and 20 nmol of each dNTP was heated for 15 min at 65°C and cooled down on ice. Ten microliters containing 200 units of SuperScript II Reverse Transcriptase (Invitrogen) in 2× first-strand buffer and 0.02 M DTT was added, and the mixture was incubated at 42°C for 30 min. Reaction was stopped by adding 10 µl containing 0.9 M LiCl, 40 µg of glycogen and 10 µg of RNase. After 5 min at room temperature, the DNA was ethanol-precipitated, centrifuged, air-dried, dissolved in 6 µl of formamide-containing loading buffer and electrophoresed in a 6% denaturing polyacrylamide gel. Sequence of primers is given in Supplementary Table S3.

### Protein analysis

Protein extracts were prepared using mechanical breakage with glass beads as described in Dunn and Wobbe (33). One microgram of total protein was separated by 12% sodium dodecyl sulphate-polyacrylamide gel electrophoresis and transferred to nitrocellulose. Green fluorescent protein (GFP) was visualized using mouse anti-GFP monoclonal antibodies (Roche) and the SuperSignal West Pico chemiluminescence detection system (Thermo Scientific).

## RESULTS

### Experimental system

**Figure 1.**
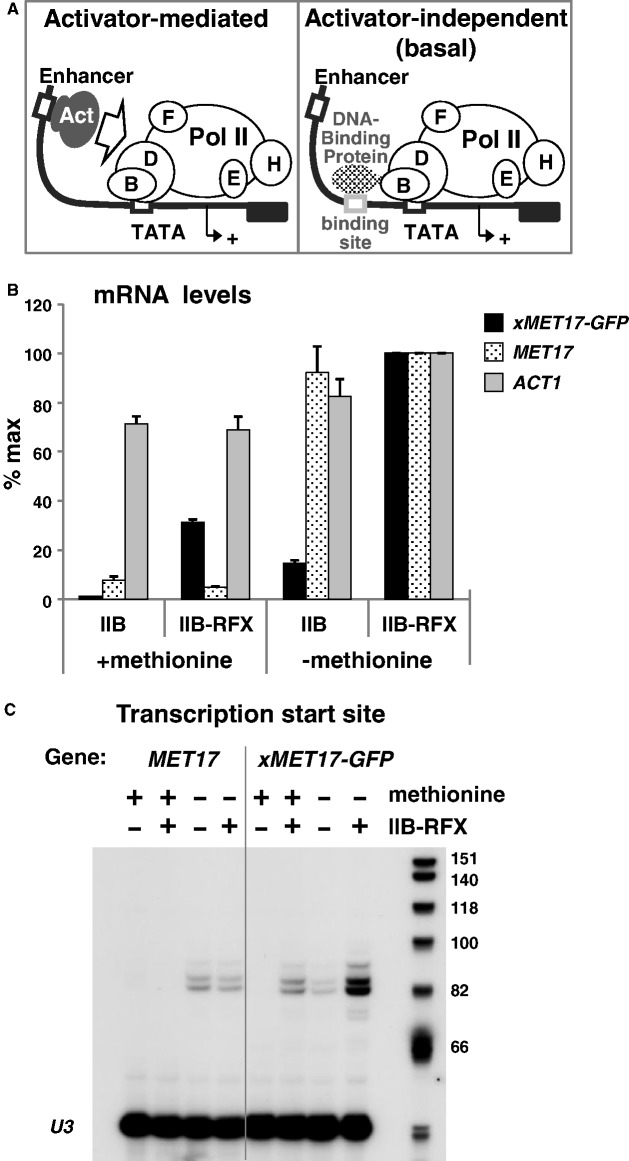
TFIIB-RFX supports accurate transcription initiation from a *MET17* promoter containing an upstream RFX binding site. (**A**) Experimental strategy. See text for details. (**B**) Strain Y892 (see Supplementary Table S1) containing YCp33-*xMET17-GFP*, and either pRS314-TFIIB (IIB) or pRS314-TFIIB-RFX (IIB-RFX), were grown in glucose-containing CSM medium supplemented with 0.5 mM methionine (+methionine), or in glucose-containing YNB medium with no methionine (−methionine). RNA levels for *xMET17-GFP*, *MET17* and *ACT1* were quantified by RT-qPCR and normalized to *25S* rRNA levels. Values are expressed as a percentage of the maximum value. Error bars represent standard deviations from three independent experiments. (**C**) Same RNA preparations as in (B) were subjected to primer extension analysis with primers for *MET17* and *xMET17-GFP* starting in both cases at exactly the same distance from *MET17* TATA box (175 bp). U3 snoRNA was used as a control.

To assess the role of Mediator in basal transcription *in vivo*, we took advantage of molecular tools allowing artificial recruitment of the Pol II machinery to a test promoter in the absence of classical activator protein in yeast (see Figure 1A). We first used the system developed by Strubin *et al.*, in which TFIIB is fused to RFX, a human sequence-specific DNA binding protein with no activation potential in yeast (34,35). The RFX binding site was introduced at 73 bp upstream of the unique TATA element present in the promoter of the methionine biosynthetic gene *MET17*. Transcription of *MET17* is induced by the activator Met4 in the absence of methionine; however, under conditions of excess of methionine, Met4 activity is downregulated through a mechanism involving degradation by the ubiquitin–proteasome system, and as a result, *MET17* transcription is shut down (36,37). *MET17* is also induced by cadmium, which inactivates the ubiquitin-ligase targeting Met4 even in the presence of excess of methionine (38). A plasmid bearing this test promoter (named as *xMET17*) fused to the coding sequence of the GFP was introduced, with a plasmid bearing TFIIB or TFIIB-RFX, into a strain containing the chromosomal TFIIB allele under control of the glucose-repressed *GAL1* promoter to shut down expression of the endogenous TFIIB. As expected, the *xMET17* promoter remained inactive in cells expressing TFIIB in the presence of 0.5 mM methionine, and attaching TFIIB to RFX led to a strong increase in *xMET17-GFP* transcription (Figure 1B). We confirmed through primer extension analysis that transcription driven by TFIIB-RFX was initiated at the same positions as transcription driven by Met4 (Figure 1C). Moreover, inactivation of Met4 rendered cells auxotroph for blocked transcriptional induction of the endogenous *MET17* genes by cadmium but had no effect on activation of *xMET17-GFP* by TFIIB-RFX (Supplementary Figure S1). Therefore, artificial tethering of TFIIB to *MET17* results in efficient and accurate transcription initiation in the absence of the activator.

### Tethering TFIIB to the *xMET17* promoter leads to recruitment of Mediator

**Figure 2.**
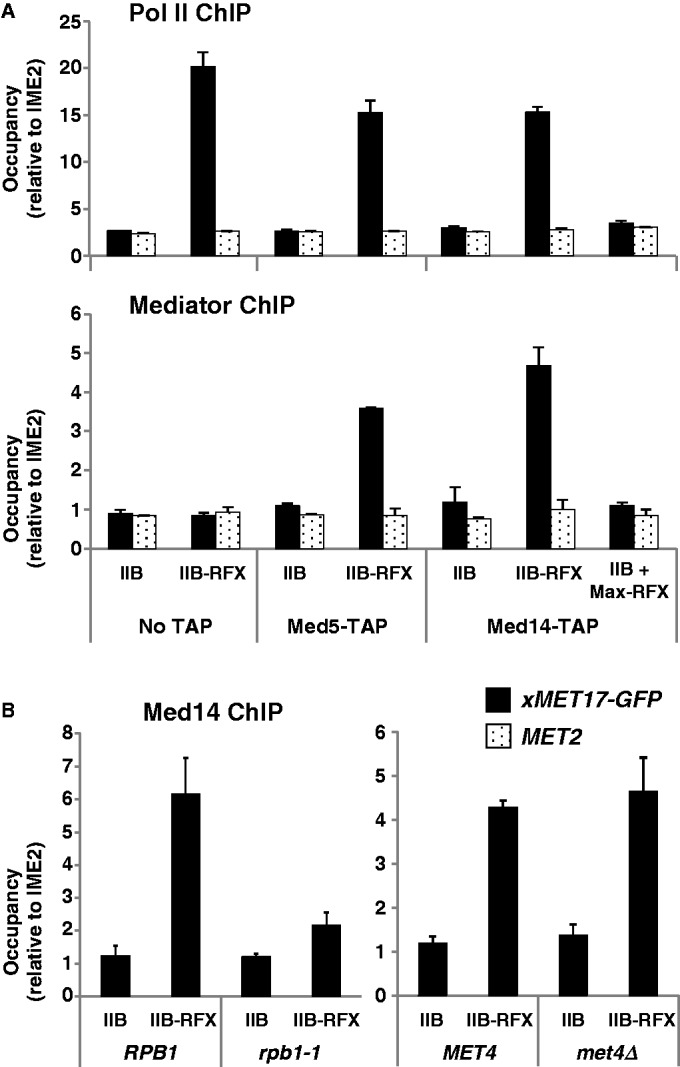
Transcriptional activation of *xMET17* by TFIIB-RFX leads to Mediator recruitment. (**A**) Untagged (No TAP) and TAP-tagged cells (Y892, Y893 and Y894) containing YCp33-*xMET17-GFP* and either pRS313-IIB or pRS313-IIB-RFX, or pRS313-IIB plus pRS315-Max-RFX, were grown in glucose-containing CSM medium supplemented with 0.5 mM methionine. Mediator and Pol II occupancy at *xMET17-GFP* and *MET2* was measured by ChIP. DNA was quantitated by qPCR using primers specific for the ORF (Pol II ChIP) or for the promoter (Mediator ChIP). Occupancy levels were normalized using the ORF of the transcriptionally inactive gene *IME2*. Error bars indicate standard deviations from three independent experiments. (**B**) Left graph: A Med14 TAP-tagged *rpb1-1* mutant and the isogenic wild-type strain (Y909 and Y911) containing YCp33-*xMET17-GFP* and pRS313-IIB or pRS313-IIB-RFX, were grown to early log phase at 25°C in CSM medium supplemented with 0.5 mM methionine, and were shifted to 37°C for 45 min before formaldehyde fixation. Mediator occupancy was measured by ChIP as in (A). Right graph: A *met4*-disrupted, Med14 TAP-tagged strain (*met4Δ*) and the isogenic wild-type strain (Y894 and Y963) containing YCp33-*xMET17-GFP*, and pRS314-IIB or pRS314-IIB-RFX, were grown and submitted to ChIP as above.

To determine whether Mediator is present at the *xMET17* promoter when transcription is driven by TFIIB-RFX, we carried out chromatin immunoprecipitation (ChIP) experiments using cells containing TAP-tagged versions of Med5 and Med14, two Mediator subunits belonging to the middle and tail modules, respectively. The results for *Med14-TAP* and *Med5-TAP* showed a 3- to 4-fold increase in occupancy at the *xMET17* promoter in the strains expressing TFIIB-RFX compared with the strains expressing TFIIB (Figure 2A, bottom). In contrast, no increase of occupancy was observed in the untagged strains or at the promoter of *MET2*, another Met4-regulated gene not activated in the presence of methionine. As expected, Pol II occupancy at *xMET17* was similar in the tagged and untagged strains expressing TFIIB-RFX (Figure 2A, top). To ascertain that Mediator recruitment occurred in association with the Pol II machinery and not through interaction with the RFX moiety, we also used *Med14-TAP* cells expressing RFX fused to the helix-loop-helix-leucine zipper dimerization motif present in the human Max oncogene (25). Expression of Max-RFX did not lead to any increase in Med14 occupancy at *xMET17* (Figure 2A, bottom), indicating that the RFX moiety is not able to recruit Mediator. In parallel, we checked Mediator occupancy at *xMET17* in cells containing the *rpb1-1* temperature-sensitive mutation within Pol II, which causes a genome-wide transcriptional arrest at 37°C (6). The results showed that Med14 occupancy was 3-fold lower in the *rpb1-1* mutant compared with the wild-type cells after 45 min at 37°C (Figure 2B, left graph). Finally, to completely rule out any participation of *Met4*, *Med14-TAP* occupancy was also assayed in the *met4Δ* strain containing a null allele of the *MET4* gene. The results showed no effect of *MET4* deletion on *Med14-TAP* association with *xMET17* (Figure 2B, right graph). We concluded from all these results that Mediator can be recruited to *xMET17* in cells containing TFIIB-RFX by virtue of its ability to interact with Pol II and its GTFs.

### Transcriptional activation by TFIIB-RFX is not affected in mutants of Mediator tail module

**Figure 3.**
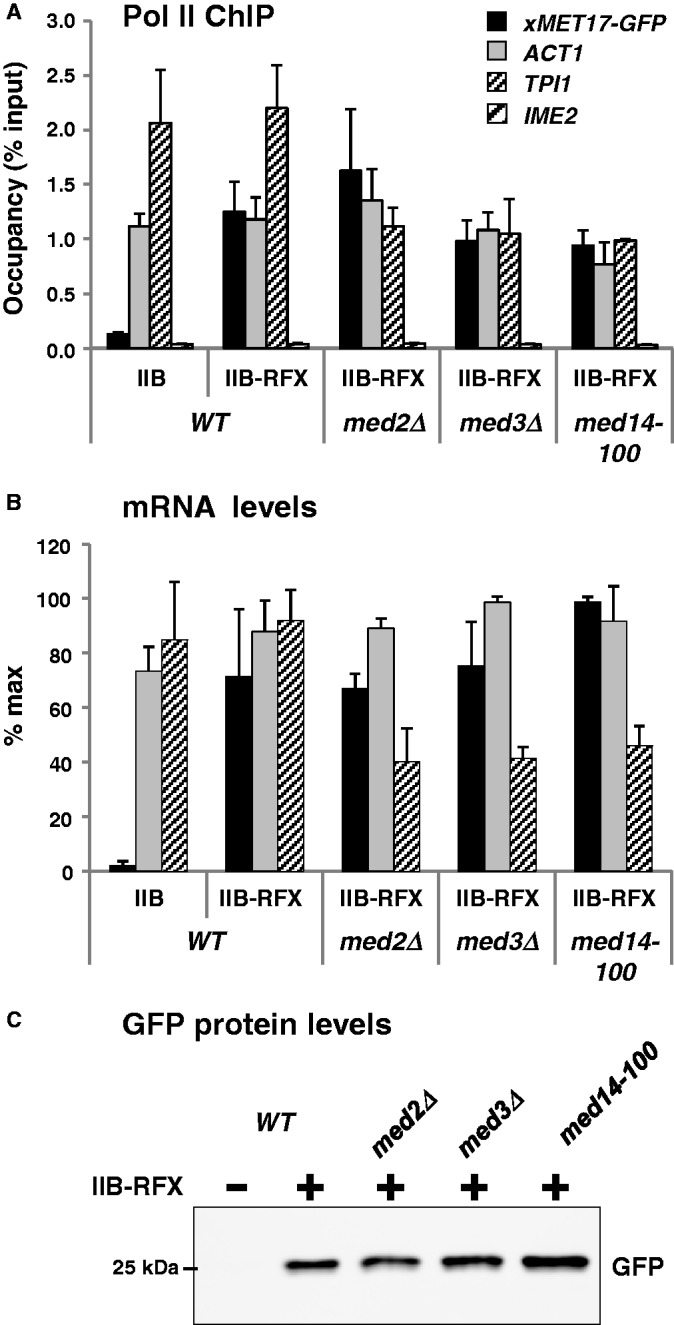
Mutation of Mediator tail module has no effect on transcriptional activation of *xMET17* by TFIIB-RFX. The *med2Δ*, *med3 Δ* and *med14-100* mutants (Y807, Y809 and Y811) and the isogenic wild-type strain (Y805) containing YCp33-*xMET17-GFP*, and either pRS314-IIB or pRS314-IIB-RFX, were grown at 28°C in glucose-containing CSM medium supplemented with 0.5 mM methionine. (**A**) Pol II occupancy at *xMET17-GFP*, *ACT1*, *TPI1* and *IME2* was measured by ChIP. DNA was analysed by qPCR using primers for the ORFs of *GFP* (5′ORF), *ACT1, TPI1* and *IME2*. Error bars indicate standard deviations from three independent experiments. (**B**) RNA levels quantified as in Figure 1. (**C**) Western blot on whole cell extracts using anti-GFP antibody.

Mediator tail module is viewed as the main interface through which transcriptional activators come into contact with the Mediator. Accordingly, we showed in our previous report that transcriptional activation of *Met4*-dependent genes, including *MET17*, is severely affected by deletion of the non-essential tail subunits Med2 and Med3, or by a C-terminal truncation in the essential tail subunit Med14 (also known as Rgr1) (27). Therefore, we asked whether the same mutations would also affect transcriptional activation of *xMET17-GFP* by TFIIB-RFX. The results showed that the *med2Δ*, *med3Δ* and *med14-100* mutations did not lead to any significant defects in Pol II occupancy at *xMET17* (Figure 3A), and they affected neither *xMET17-GFP* transcript levels (Figure 3B) nor GFP protein levels (Figure 3C). As already reported (39), transcription of *ACT1* was not affected in the mutants, whereas transcription of *TPI1* was decreased by 2-fold. These results are consistent with the observation that inactivation of Med2 or Med3 affects only a limited subset of genes (40), which suggests no essential role in assembly and activity of the Pol II machinery.

### Med17 is required for transcriptional activation by TFIIB-RFX

**Figure 4.**
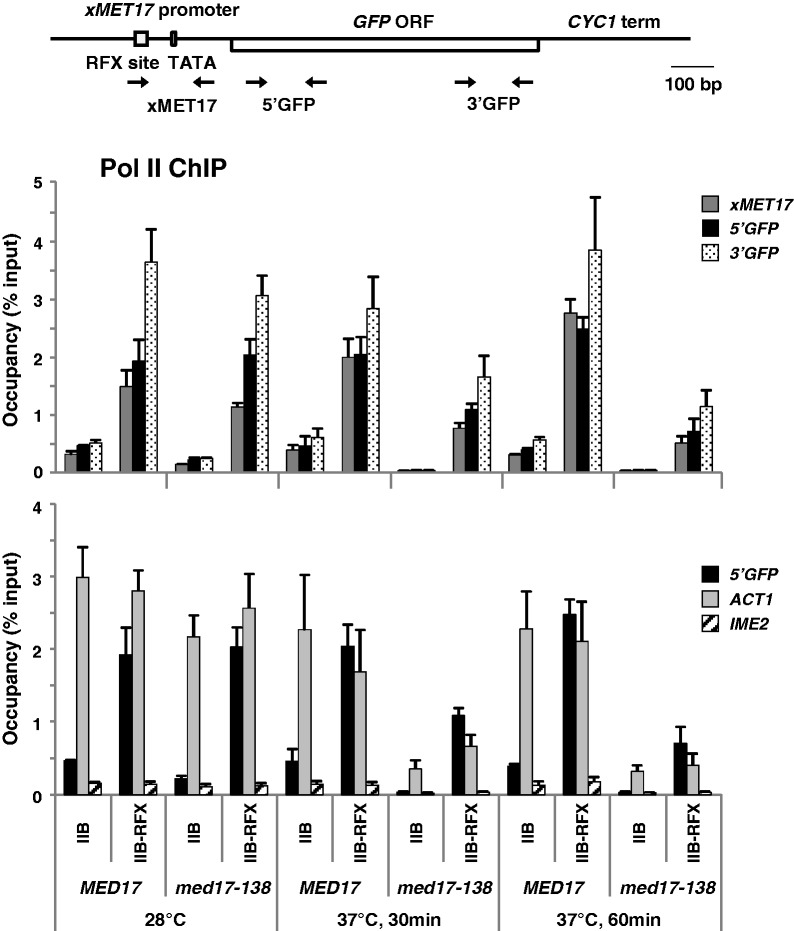
Med17 inactivation affects transcriptional activation by TFIIB-RFX from the *xMET17* promoter. The *med17-138* mutant and an isogenic wild-type strain (Y822 and Y823) containing YCp33-*xMET17-GFP*, and either pRS314-IIB or pRS314-IIB-RFX, were grown at 28°C to early log phase in glucose-containing CSM medium supplemented with 0.5 mM methionine, and were shifted at 37°C. Pol II occupancy was analysed by ChIP. Samples were fixed with formaldehyde before, and 30 or 60 min after the temperature shift. DNA was analysed by qPCR using primers for the promoter or the ORF of *xMET17-GFP* (see top schematic diagram), and primers for the ORF of *ACT1* and *IME2*. Error bars indicate standard deviations from four independent experiments.

We next used a mutation in the Med17 subunit located at the Mediator head module, knowing that this module makes multiple contacts with Pol II in the structural models (4,41). Med17 has recently been shown to interact directly with Pol II *in vivo* (42). We used the well-characterized *med17-138* temperature-sensitive mutant, which ceases transcription of all mRNA in a manner similar to the *rpb1-1* mutant at 37°C (6). Mutant and isogenic wild-type cells expressing TFIIB-RFX showed similar levels of Pol II occupancy at *xMET17-GFP* at 28°C (Figure 4). In contrast, occupancy was decreased 3- to 4-fold in the mutant compared with the wild-type cells 60 min after transfer to 37°C. Importantly, a similar decrease was observed with primers centered on the TATA box of *MET17*, and primers positioned at the beginning or at the end of the ORF of *GFP* (Figure 4, top graph), which indicates a defect in Pol II recruitment to the promoter and not a defect in promoter clearance or elongation. Consistently, the decrease in Pol II association was accompanied by a similar decrease in mRNA levels (Supplementary Figure S2).

For comparison, ChIP experiments were also performed with strains carrying a temperature-sensitive mutation in the kinase subunit of TFIIH. We used the *kin28-ts3* mutation already known to cause a global shutdown of mRNA synthesis at 37°C (6,30). The mutant and the wild-type cells expressing TFIIB-RFX showed similar levels of Pol II occupancy at *xMET17-GFP* at the permissive temperature (25°C; Supplementary Figure S3). However, occupancy was decreased by 3- to 4-fold in the mutant cells compared with the wild-type cells at 37°C. Therefore, inactivation of Med17 and Kin28 has the same effect on transcriptional activation of *xMET17-RFX* by TFIIB-RFX.

**Figure 5.**
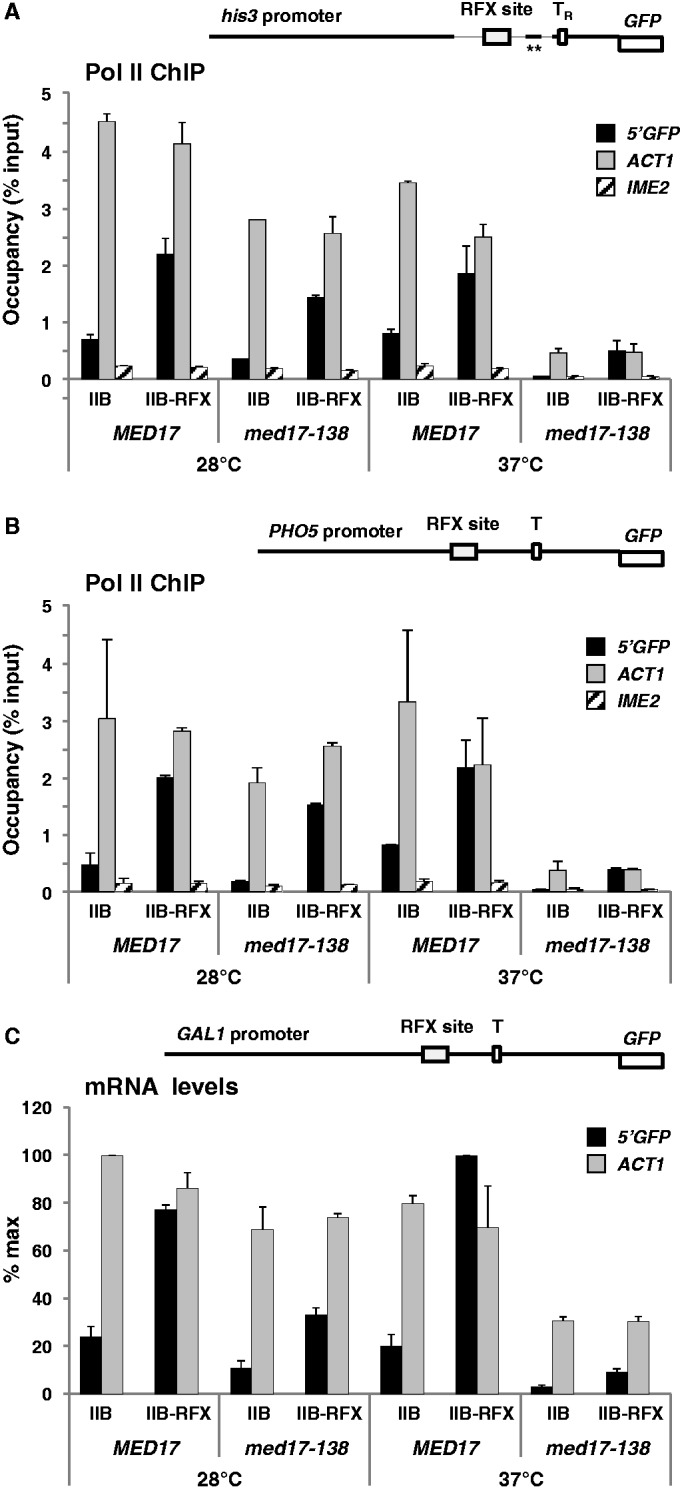
Transcriptional activation by TFIIB-RFX from *HIS3*, *PHO5* and *GAL1* promoter derivatives containing the RFX binding site is affected in the *med17-138* mutant. (**A**) Top diagram: schematic representation of the *xhis3-GFP* test gene containing an RFX binding site 72 bp upstream the canonical TATA element (T_R_) of *HIS3*. The thick lines corresponds from left to right to positions −750 to −464, −125 to −108 and −78 to −1 of *HIS3*, and the asterisks indicate mutations in the Gcn4 binding site. T_R_ is at −70. Graph: Pol II occupancy at *GFP*, *ACT1* and *IME2* ORF measured by ChIP. The *med17-138* mutant and an isogenic wild-type strain (Y822 and Y823) containing YCp33-*xhis3-GFP*, and either pRS314-IIB or pRS314-IIB-RFX, were grown in glucose-containing CSM medium at 28°C to early log phase, and were shifted to 37°C. Samples were processed for ChIP analysis as in Figure 4. Error bars represent standard deviations from two independent experiments. (**B**) Upper diagram: schematic representation of the *xPHO5-GFP* gene containing an RFX binding site 80 bp upstream *PHO5* TATA element. The thick line corresponds to positions −392 to −1 of *PHO5*. The TATA element is at −101. Graph: Pol II occupancy at *GFP*, *ACT1* and *IME2* ORF measured by ChIP. The *med17-138* mutant and an isogenic wild-type strain (Y822 and Y823) containing YCp33-*xPHO5-GFP*, and either pRS314-IIB or pRS314-IIB-RFX, were grown and processed for ChIP analysis as in (A). (**C**) Top diagram: schematic representation of the *xGAL1-GFP* gene containing an RFX binding site 67 bp upstream of the *GAL1* TATA element. The thick line corresponds to positions −500 to −1 of *GAL1*. The TATA element is at −147. Graph: RNA levels for *xGAL1-GFP* and *ACT1* quantified by RT-qPCR as shown in Figure 1. Error bars represent standard deviations from two independent experiments.

To validate further our findings, we used three additional test promoters containing the RFX binding site inserted within *HIS3*, *PHO5* or *GAL1* promoters (Figure 5). *HIS3* is regulated by the activator of the general amino acid control Gcn4. The *HIS3* promoter possesses two TATA elements: a T_R_ element that contains a canonical TATA sequence and is responsible for transcription from the +13 initiation site, and a more upstream T_C_ element that lacks a conventional TATA sequence and is responsible for transcription from the +1 site (43). The *his3* derivative used here is devoid of the T_C_ element and carries two mutations that completely inactivate the binding site for Gcn4 (44) (see diagram in Figure 5A). *PHO5* and *GAL1* are two tightly regulated promoters: *PHO5* is not expressed under high-phosphate conditions because of exclusion of the activator Pho4 from the nucleus (45); *GAL1* is not expressed in the absence of galactose because of inhibition of the activator Gal4 by the repressor Gal80, which binds Gal4 activation domain and prevents recruitment of the Pol II machinery, including Mediator (46). *PHO5* and *GAL1* both contain a unique conventional TATA element (see diagram in Figure 5B and C). The results in Figure 5 and Supplementary Figure S4 show that attaching TFIIB to RFX led to an increase in Pol II recruitment (Figure 5A and B) and transcription (Figure 5C and Supplementary Figure S4) for all three genes (note that only RNA levels, not Pol II recruitment, were assessed for *xGAL1-GFP* because transcriptional activation was too weak to give ChIP signals over background). Moreover, as in the case of *xMET17-GFP*, inactivation of Med17 resulted in a decrease in transcription activation of the three promoters by TFIIB-RFX (Figure 5 and Supplementary Figure S4). For instance, Pol II occupancy at *xhis3-GFP* and *xPHO5-GFP* was decreased by 4- and 6-fold, respectively, in the *med17-138* mutant compared with the wild-type cells after 60 min at 37°C (Figure 5A and B), and mRNA levels for *xGAL1-GFP* were decreased >10-fold (Figure 5C).

**Figure 6.**
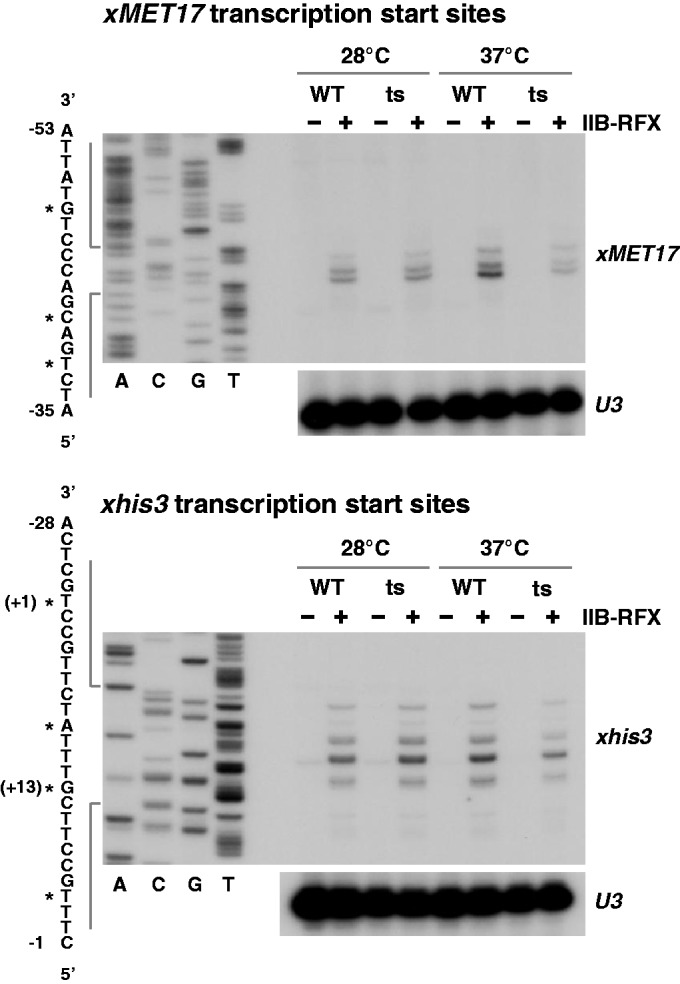
Med17 inactivation has no effect on start site selection at *xMET17* and *xhis3* promoters. Primer extension analysis was performed using the same RNA preparations as shown in Figure 4 (*xMET17* start sites) and Figure 5 (*xhis3* start sites) with a primer starting at position +74 of *GFP*. The sequencing DNA ladder was generated using the same primer and a plasmid bearing *xMET17-GFP* or *xhis3-GFP*. The asterisks indicate the major TSSs. Positions are given relative to the start codon. *U3* snoRNA was used as a control.

Primer extension analysis was also performed to assess whether Med17 inactivation had an effect on transcription start site (TSS) selection. We analysed TSS positions at *xMET17* and *xhis3*, which both possess multiple TSSs (Figure 6). The results showed a parallel decrease in transcription initiated at all sites in both cases, and no obvious change in positions (Figure 6). Therefore, inactivation of Med17 leads to a decrease in transcriptional activation by TFIIB-RFX, but does not change the position of the TSSs.

### Transcription activation by artificial tethering of TBP also depends on Mediator head module

**Figure 7.**
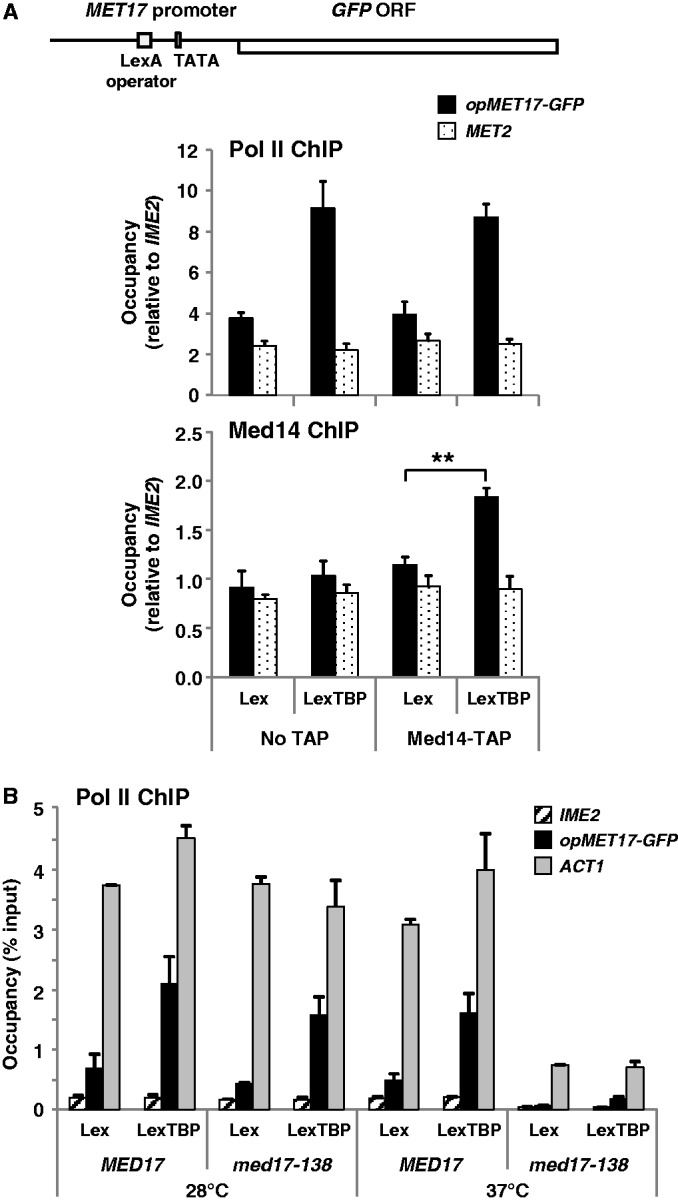
Transcriptional activation by LexA-TBP from a *MET17* derivative containing the LexA operator requires Mediator. (**A**) Untagged (No TAP) and Med14 TAP-tagged cells (Y14 and Y84) containing YCp33-op*MET17-GFP* (see schematic representation) and either pRS313-LexA (Lex) or pRS313-LexA-TBP (LexTBP) were grown as shown in Figure 2. Pol II and Med14-TAP occupancy at *opMET17-GFP* and *MET2* was measured by ChIP. DNA was analysed by qPCR using primers for the ORF (Pol II ChIP) or the promoter (Mediator ChIP). Occupancy levels were normalized using *IME2* ORF. Error bars indicate standard deviations from three (Pol II ChIP) or four (Mediator ChIP) independent experiments. Asterisks indicate *P* < 0.005 in a Student’s *t* test. (**B**) The *med17(srb4)-138* mutant and an isogenic wild-type strain (Y400 and Y402) containing YCp33-op*MET17-GFP* and either YCp91-LexA (Lex) or YCp91-LexA-TBP (LexTBP) were grown at 28°C to early log phase in CSM medium supplemented with 0.5 mM methionine, and were shifted to 37°C. Pol II occupancy was measured by ChIP before and 60 min after the shift. DNA was analysed by qPCR using primers for the 5′-end of *GFP* ORF, *ACT1* ORF and *IME2* ORF. Error bars indicate standard deviations from two independent experiments.

Finally, we asked whether Mediator is also required when the Pol II machinery is recruited to DNA by tethering TBP instead of TFIIB. To this end, we used a LexA-TBP hybrid containing TBP fused to the full-length bacterial repressor LexA (24), and a *LexAop-MET17-GFP* test reporter containing one LexA operator inserted 69 bp upstream of the TATA box of *MET17* (*see* schematic in Figure 7A). ChIP experiments were carried out using Med14 TAP-tagged strains. The results showed a modest (1.8-fold) but significant increase in Med14 occupancy at *LexAop-MET17* in the TAP-tagged strain expressing LexA-TBP compared with the TAP-tagged strain expressing LexA alone (Figure 7A, bottom graph). This low occupancy is consistent with the fact that LexA-TBP led to lower levels of Pol II recruitment than TFIIB-RFX. ChIP experiments were also carried out using the *med17-138* mutant and the isogenic wild-type strain. As shown in Figure 7B, mutant and isogenic wild-type cells expressing LexA-TBP showed similar levels of Pol II occupancy at *LexAopMET17-GFP* at 28°C. In contrast, occupancy was decreased 9-fold in the mutant compared with the wild type cells after 60 min at 37°C. Therefore, transcription activation by tethering TBP to the promoter is also accompanied by recruitment of Mediator and is also sensitive to inactivation of Med17.

## DISCUSSION

### Mediator recruitment can occur independently of activator

Conventional and genome-wide ChIP analyses in yeast have clearly demonstrated that Mediator is crosslinked by formaldehyde preferentially to enhancers rather than to core promoters where Pol II binds; moreover, based on time-course experiments and mutant studies, it was also shown that Mediator association with promoters is independent of Pol II, GTFs and core promoter sequences (7–9,20,21,47). Altogether, these studies support the view that the recruitment of Mediator to promoters occurred through activators bound to enhancers. We show here that Mediator can also be recruited to promoters *in vivo* independently of the presence of an activator, solely via its ability to interact with Pol II and the GTFs. Mediator has been known for a long time to form a biochemically stable complex with Pol II (16,48), and physical contacts with GTFs such as TBP and TFIIH were also shown (15,49). The demonstration of a direct interaction between individual subunits of Pol II and Mediator *in vivo* has also been reported recently (42) but, to our knowledge, this study provides the first demonstration that the recruitment of Mediator to a promoter can occur *in vivo* in the absence of interaction with an activator. This scarcity of data is certainly a consequence of the transient nature of the interaction between Mediator and Pol II at promoters, which occurs only during transcription initiation and must be broken to allow Pol II escape (50), whereas the interaction between Mediator and activators is more stable and persists after Pol II has left the promoter (9,51).

### Mediator head is required for activator-independent transcription *in vivo*

Our results with *med17-138* demonstrate a role of Mediator in assembly of the basal Pol II initiation machinery at promoters even in the absence of signals transmitted by activators. Importantly, this conclusion was reached using two GTFs tethered to four different test promoters through two different DNA binding proteins. Strikingly, inactivation of Med17 has a more pronounced effect on Pol II recruitment to *opMET17* via LexA-TBP than on Pol II recruitment to *xMET17* via TFIIB-RFX (compare Figures 4 and 7). This result suggests that tethering of TFIIB to the promoter can, at least partly, compensate for the loss of Med17, thus pointing toward a role of Mediator in stabilizing TFIIB within the PIC *in vivo*. This view is in line with a previous *in vitro* study showing that the requirement of human Mediator for basal transcription in nuclear extracts can be alleviated by increasing the TFIIB amount (52). Part of the residual Pol II recruitment observed in the *med17-138* mutant at a restrictive temperature is also certainly owing to incomplete inactivation of Med17. Indeed, it was observed that the *MED6-101* dominant mutation could suppress the temperature-sensitive phenotype of *med17-138* but not the lethality associated with the *med17*-null allele, indicating that the Med17-138 protein retains some function even at a non-permissive temperature (53). This explains why Pol II occupancy remains higher at *ACT1* than at *IME2* in *med17-138* after 60 min at a non-permissive temperature (Figures 4 and 7).

In contrast with Med17, we have found that inactivation of subunits of the tail module does not affect activation of the *xMET17* promoter by TFIIB-RFX, even though this module is required for activation of *MET* genes by Met4 (27). In agreement with the idea that the tail module serves mainly as an interface for activators, our results provide *in vivo* confirmation to the finding that Mediator head alone is sufficient to stimulate basal transcription *in vitro* (41,54).

### Mediator association with Pol II *in vivo* does not require activator-induced conformational change

Structural studies showed that the yeast Mediator complex adopts two different conformations when free or when associated with Pol II: the free complex presents a compact conformation with the middle and tail modules folded on each other, whereas the complex associated with Pol II presents an extended conformation, with Pol II binding in a pocket generated when Mediator changes conformation (55). It is not known whether this change in conformation occurs in the context of the living cell. However, if it does occur, the question remains whether it occurs spontaneously or is triggered by contact with activators. Our results argue that Mediator can be recruited to promoters and exert its function on assembly and activity of the PIC in the absence of an activator. Therefore, if any change in conformation is required, it can happen without the presence of an activator. This conclusion is in line with recent cryo-EM analyses of the human Mediator-Pol II-TFIIF assembly, which showed that Pol II binds Mediator at the same general location in the presence or the absence of an activator (13,14).

Mediator is often presented as a relay conveying regulatory signals from gene-specific activators bound at distal regulatory elements to the basal Pol II machinery located at the core promoter. The present study shows that the yeast Mediator functions not only through targeted recruitment by gene-specific factors, but also in an untargeted manner, strongly supporting a model in which Mediator is an integral part of the basic Pol II initiation machinery and can exert a function independently of regulatory signals conveyed by activators. Considering the functional and structural conservation of Mediator (2,56), this model should apply to all eukaryotes.

## SUPPLEMENTARY DATA

Supplementary Data are available at NAR Online.

## FUNDING

Centre National de la Recherche Scientifique, and grants from Agence Nationale pour la Recherche [ANR-06-JCJC-0045 and ANR-08-BLAN-0229]; and Association pour la Recherche sur le Cancer [subvention 4901]. Funding for open access charge: Centre National de la Recherche Scientifique.

*Conflict of interest statement*. None declared.

## Supplementary Material

Supplementary Data
