# Supplementary material for: Mediator is an intrinsic component of the basal RNA polymerase II machinery in vivo
Source: Nucleic Acids Res. 2013 Aug 20;41(21):9651–62. doi: 10.1093/nar/gkt701 (PMC3834807; doi:10.1093/nar/gkt701)
Supplement: Supplementary Data [file supp_41_21_9651__index.html]

Mediator is an intrinsic component of the basal RNA polymerase II machinery in vivo — Mediator is an intrinsic component of the basal RNA polymerase II machinery in vivo — Supplementary Data 

# Mediator is an intrinsic component of the basal RNA polymerase II machinery *in vivo*

## Supplementary Data

files

**Files in this Data Supplement:**

- Supplementary Data - pdf file
