# Supplementary material for: Mediator is an intrinsic component of the basal RNA polymerase II machinery in vivo
Source: Nucleic Acids Res. 2013 Aug 20;41(21):9651–62. doi: 10.1093/nar/gkt701 (PMC3834807; doi:10.1093/nar/gkt701)
Supplement: Supplementary Data [file supp_gkt701_nar-00448-r-2013-File009.pdf]

Supplementary material

Mediator is an intrinsic component of the basal RNA polymerase II machinery *in vivo*

Thierry Lacombe, Siew Lay Poh, Régine Barbey , and Laurent Kuras.

Table S1. *Saccharomyces cerevisiae* strains.

Strain	Relevant genotype	Source
Y14	*MAT*a *ade2 ura3 his3 leu2 trp1 SPT15*::*3HA-SPT15*	Kuras&Struhl (1999)
Y84	*MAT*a *ade2 ura3 his3 leu2 trp1 RGR1*::*RGR1-TAP-KlTRP1 SPT15*::*3HA-SPT15*	Kuras *et al* (2003)
Y400	*MAT*a *ade2 ura3 his3 leu2 trp1 srb4*::*His3MX6* [RY2844 (*SRB4*, *LEU2*, *CEN*)]	This study
Y402	*MAT*a *ade2 ura3 his3 leu2 trp1 srb4*::*His3MX6* [RY2882 (*srb4-138*, *LEU2*, *CEN*)]	This study
Y805	*MAT*a *ade2 ura3 his3 leu2 trp1 SUA7*::*KanMX6-PGAL1-SUA7*	This study
Y807	*MAT*a *ade2 ura3 his3 leu2 trp1 med2Δ*::*His3MX6 SUA7*::*KanMX6-PGAL1-SUA7*	This study
Y809	*MAT*a *ade2 ura3 his3 leu2 trp1 med3Δ*::*His3MX6 SUA7*::*KanMX6-PGAL1-SUA7*	This study
Y811	*MAT*a *ade2 ura3 his3 leu2 trp1 lys2 rgr1-100 SUA7*::*KanMX6-PGAL1-SUA7*	This study
Y822	*MAT*a *ade2 ura3 his3 leu2 trp1 srb4*::*His3MX6 SUA7*::*KanMX6-PGAL1-SUA7* [RY2844 (*SRB4*, *LEU2*, *CEN*)]	This study
Y823	*MAT*a *ade2 ura3 his3 leu2 trp1 srb4*::*His3MX6 SUA7*::*KanMX6-PGAL1-SUA7* [RY2882 (*srb4-138*, *LEU2*, *CEN*)]	This study
Y892	*MAT*a *ade2 ura3 his3 leu2 trp1 SPT15*::*3HA-SPT15 SUA7*::*KanMX6-PGAL1-SUA7*	This study
Y893	*MAT*a *ade2 ura3 his3 leu2 trp1 NUT1*::*NUT1-TAP-KlTRP1 SPT15*::*3HA-SPT15 SUA7*::*KanMX6-P_{GAL1}-SUA7*	This study
Y894	*MAT*a *ade2 ura3 his3 leu2 trp1 RGR1*::*RGR1-TAP-KlTRP1 SPT15*::*3HA-SPT15 SUA7*::*KanMX6-PGAL1-SUA7*	This study
Y909	*MAT*a *ade2 ura3 his3 leu2 trp1* RPB1 *RGR1*::*RGR1-TAP-KlTRP1 SUA7*::*KanMX6-PGAL1-SUA7*	This study
Y911	*MAT*a *ade2 ura3 his3 leu2 trp1 rpb1-1 RGR1*::*RGR1-TAP-KlTRP1 SUA7*::*KanMX6-PGAL1-SUA7*	This study
Y959	*MATα ura3 his3 leu2 trp1* SUA7::*kanMX6-PGAL1-SUA7*	This study
Y960	*MATα ura3 his3 leu2 trp1 kin28-ts3* SUA7::*kanMX6-PGAL1-SUA7*	This study
Y963	*MAT*a *ade2 ura3 his3 leu2 trp1 met4Δ::TRP1 RGR1*::*RGR1-TAP-KlTRP1 SUA7*::*KanMX6-PGAL1-SUA7*	This study

Table S2. Sequence of primers used in RT-qPCR and ChIP experiments

Gene		position	Sequence 5'->3'
25s rDNA	Fwd	+624	GGTTATATGCCGCCCGTCTTGA
	Rev	+800	CCCAACAGCTATGCTCTTACTC
ACT1 ORF	Fwd	+306	ATTATATGTTTAGAGGTTGCTGCTTTGG
	Rev	+590	CAATTCGTTGTAGAAGGTATGATGCC
5'*GFP* ORF	Fwd	+26	CTGGTGTTGTCCCAATTTTGGTTG
	Rev	+215	GCAAAACATTGAACACCATAACCGA
3'*GFP* ORF	Fwd	+520	GGTTCTGTTCAATTAGCTGACCATT
	Rev	+704	TCATCCATACCATGGGTAATACCAG
IME2 ORF	Fwd	+1173	ATCCCAAGTAGACGCAAGAGGCAAT
	Rev	+1377	TTCTTGATTTAATGTTGGTGAGCACA
MET2 promoter	Fwd	-395	ATTTCTTGCTATTGTTAGTGGCTCC
	Rev	-242	GGTGTGTGCCAAATCCAAACGATTA
MET2 ORF	Fwd	+205	GTAATTTGTCATGCCTTGACTGGGTC
	Rev	+379	ATCTAACGCCCGTCTCCTCATTTAT
MET16 ORF	Fwd	+324	GGATGGATGTGAATCGGAGG
	Rev	+443	TTGTAGGCACGATGTGCAGG
MET17 ORF	Fwd	+59	ATGCTCACAGATCCAGAGCT
	Rev	+307	TGTCACCAGTGTGTGCCAAA
xMET17	Fwd	-227	TTGCCTAGCAACTACATATGGTCACC
	Rev	-23	TGACCATATGTAGTTGCTAG
opMET17	Fwd	-129	AAGTAAAGCGTCTGTTAGAAAGGAAGTT
	Rev	+58	CAACCAAAATTGGGACAACACCAG
TPI1	Fwd	+472	ACTAACGTCGTTGTCGCTTACGAACC
	Rev	+688	AGAAACCATCGACATCAGCCTTGT

Table S3. Sequence of primers used in Primer extension experiments

Gene	Sequence 5'->3'
GFP	AACACCAGTGAATAATTCTTC (Figure 1)
	TGACCATTAACATCACCATC (Figure 6)
MET17	TTGGCCGGCGTGTAGTTGAA
U3 (*SNR17a*)	TCAAAGAGTGACGATTCCTA

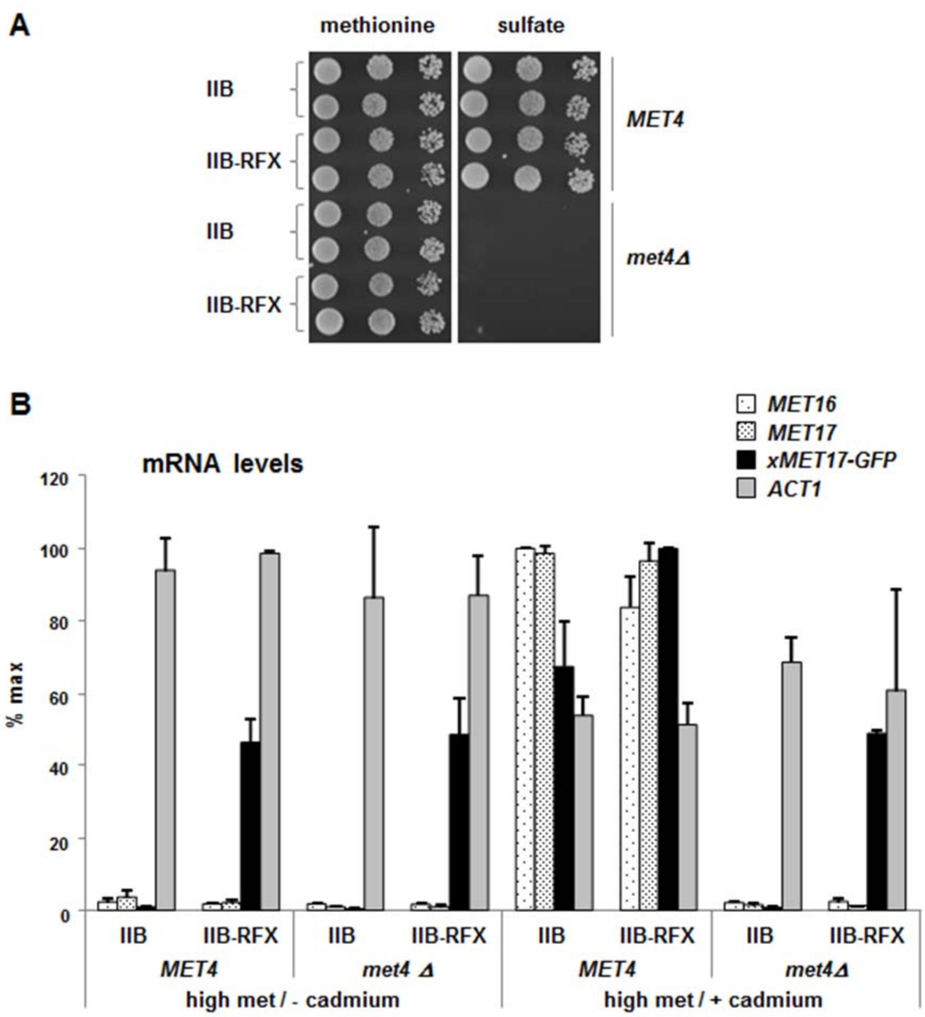

Figure S1. Effect of Met4 inactivation on transcriptional activation of *xMET17* by TFIIB-RFX. (**A**) *met4Δ* and isogenic wild-type cells (Y894 and Y963) containing YCp33-*xMET17-GFP* and either pRS313-IIB or pRS313-IIB-RFX were grown in glucose-containing CSM medium, collected, washed twice with water, and serial ten-fold dilutions were spotted on YNB medium supplemented with 0.5 mM methionine (methionine) or not (sulfate). Plates were incubated for 3 days at 28°C. (**B**) Same strains as in (A) were grown in glucose-containing CSM medium supplemented with 0.5 mM methionine in the absence (high met/- cadmium) or in the presence (high met/+ cadmium) of 0.5 mM cadmium acetate for 45 min. RNA levels were quantified by RT-qPCR as in Figure 1 using PCR primers specific for *MET16*, *MET17*, *GFP* (5'*GFP*) and *ACT1* ORF. Error bars represent standard deviations from two independent experiments.

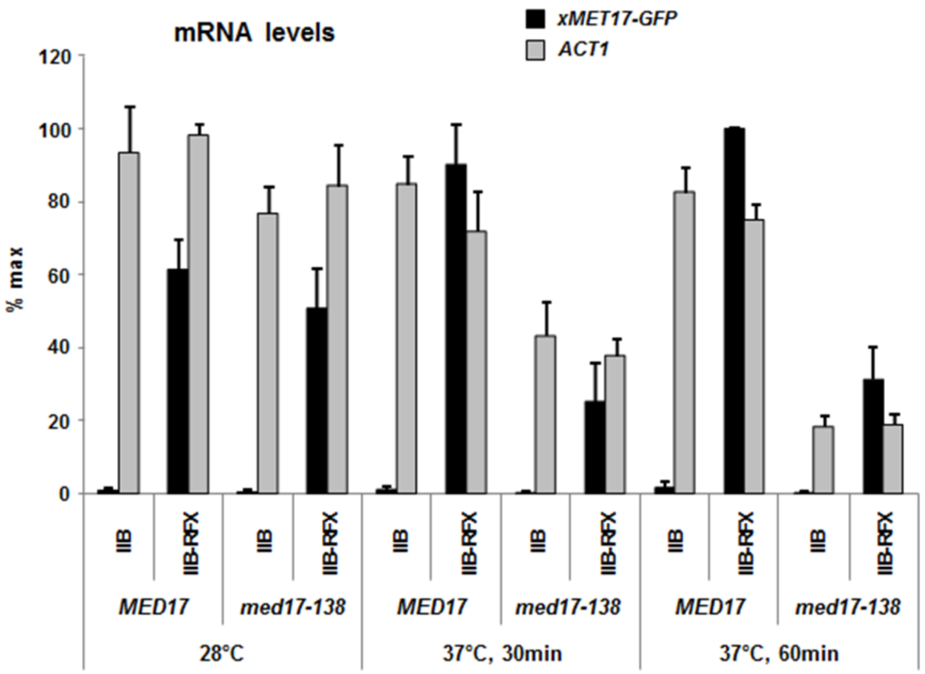

Figure S2. Med17 inactivation affects transcriptional activation by TFIIB-RFX from the *xMET17* promoter. The *med17(srb4)-138* mutant and an isogenic wild-type strain (Y822 and Y823) containing YCp33-*xMET17-GFP* and either pRS314-IIB or pRS314-IIB-RFX, were grown at 28°C in synthetic medium supplemented with 0.5 mM methionine and shifted at 37°C. RNA levels for *xMET17-GFP* and *ACT1* were quantified by RT-qPCR as in Figure 1 using PCR primers specific for the ORFs of *GFP* (*5'GFP*) and *ACT1* ORF. Error bars represent standard deviations from four independent experiments.

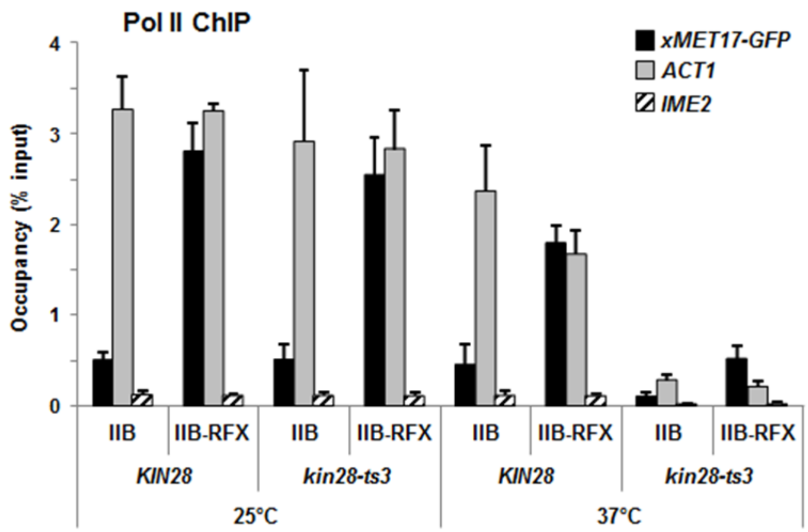

Figure S3. Effect of TFIIH inactivation on transcriptional activation by TFIIB-RFX from the *xMET17* promoter. The *kin28-ts3* temperature sensitive mutant and an isogenic wild-type strain (Y959 and Y960) containing YCp33-*xMET17-GFP* and either pRS314-IIB or pRS314-IIB-RFX were grown to early log phase at 25°C in glucose-containing CSM medium supplemented with 0.5 mM methionine, and were shifted to 37°C. Pol II occupancy was measured by ChIP before and 60 min after the shift. The cultures at 37°C were brought to 25°C before crosslinking by mixing with an equivalent volume of pre-cooled medium. DNA was analyzed by qPCR using primers specific for the ORFs of *GFP* (5' ORF), *ACT1* and *IME2*. Error bars indicate standard deviations from three independent experiments.

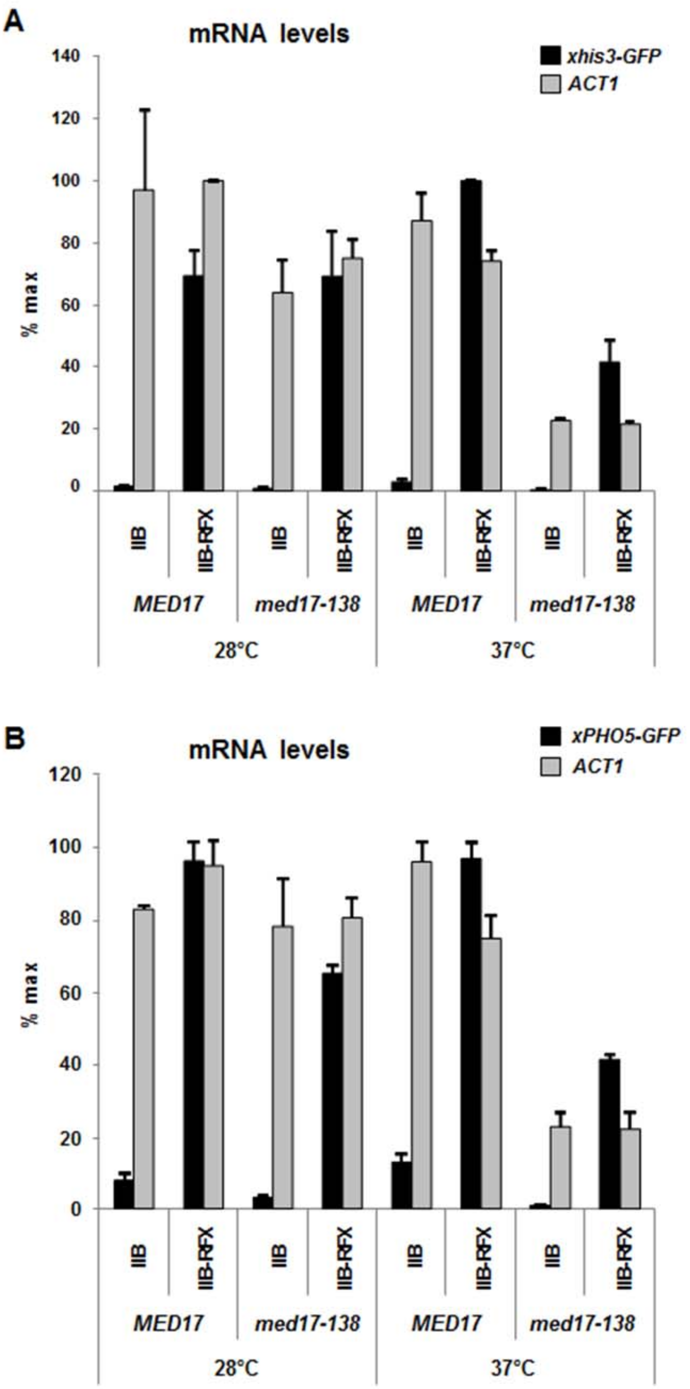

Figure S4. Med17 inactivation affects transcriptional activation by TFIIB-RFX from the *xhis3 and xPHO5* promoters. The *med17(srb4)-138* mutant and an isogenic wild-type strain (Y822 and Y823) containing YCp33-*xhis3-GFP* (**A**) or YCp33-*xPHO5-GFP* (**B**) and either pRS314-IIB or pRS314-IIB-RFX, were grown at 28°C in synthetic medium supplemented with 0.5 mM methionine and shifted at 37°C for 60 min. RNA levels for *xPHO5-GFP* and *ACT1* were quantified by RT-qPCR as in Figure 1 using PCR primers specific for the ORFs of *GFP* (*5'GFP*) and *ACT1* ORF. Error bars represent standard deviations from two independent experiments.
